# Salicine in Diarrhœa and Dysentery

**Published:** 1874-10

**Authors:** J. C. Bishop

**Affiliations:** Midddeport, Ohio


### SALICINE IN DIARRHŒA AND DYSENTERY.

BY J. C. BISHOP, M.D., OF MIDDDEPORT, OHIO.

That season of the year being at hand, when both children and adults are peculiarly susceptible to enteric affections, it may not be out of place to call the attention of the profession to the efficacy of salicine, the active principle of the *Salix Alba,* in some of these disorders. Heretofore this drug has been employed principally as a substitute for Peruvian bark in intermittent fevers, especially where the stomach rebels against the quinia, with good results. Having had occasion to observe its peculiar effects, I have lately employed it in a number of cases, not less than twenty, of diarrhœa and dysentery in both children and adults, with a success so unlooked for, that I am induced to recommend it to the notice of the profession, with the hope that it may prove equally as beneficial in the hands of others as in my own. The precise "*modus operandi*" of the drug, I shall not at this time pretend to explain; yet, that it has a peculiarly astringent and tonic action upon the mucous membrane of the stomach and intestines, seems well established by its effects, as manifested in disorders of these viscera; nor have I noticed any marked systemic effects in the cases in which it has been employed, other than those ascribed; and yet it has entirely supplanted the use of narcotics in these affections in my practice.

The following cases taken as they occurred, without reference to publication, will serve to illustrate the effects of its employment in the cases reported, which are borne out, as I am informed, in the practice of other medical gentlemen in this place.

CASE I.—C——, male, æt eight months, chronic diarrhœa; discharges almost constant, thin, watery, and emitting an intolerable odor; tenesmus extreme; abdomen tender and sympanitic; patient poorly nourished; emaciated almost to a skeleton; very weak; eyes sunken, and presenting a peculiarly pinched expression of countenance; had been sick about four weeks when I saw him; prescribed the following: ℞. Salicine, ℈i.; sach. alb., gr. x.; ft. chart, No. x. M. SIG. One powder to be given every two hours in a little milk. This was February 15, 1874.

February 16.—Saw patient again, and was surprised to find him resting well, the discharges less frequent, and general appearance slightly improved; treatment continued.

February 17.—Patient still improving; nurses with avidity, and milk seems perfectly digested; abdomen less tender and tympanitic; pulse nearly normal; respirations easy and regular.

February 18.—Patient presents appearance of comfort; diarrhœa almost entirely relieved; general condition much improved; treatment continued as a precautionary measure for a week; discharged.

CASE II.—F. Q——, male, æt two and a half, formerly vigorous and active, now languid and restless; has been suffering from extreme diarrhœa for several weeks; discharges offensive, and amounting to fifteen or twenty in the twenty-four hours; no appetite; skin hot and dry; tongue furred; bowels tender and tympanitic; prescribed same as above, and in two days patient discharged well.

CASE III.—W——, male, æt one year; thin, nervous and poorly nourished; had been suffering with diarrhœa for a week; discharges frequent, offensive, and containing considerable mucus and blood; circulation and temperature considerably increased in morning, and profuse nocturnal hydrosis; prescribed the salicine same as above, and in two days patient discharged well.

Case IV.—W. J——, male, laborer, æt fifty, bilious habit; presented a severe type of dysentery; discharges quite frequent, scanty and containing mucus and blood; tenesmus extreme; had been sick four days; no appetite; skin hot and dry; tongue coated and protruded with difficulty; complains of great pain in bowels, increased on presure; patient weak and irritable; symptoms tending to increase in severity towards evening; prescribed: Salicine, ℥i; ft. chart, No. x. Sig. One powder to be given every three hours. This was July 14, 1874.

July 15.—Complains of slight tinitus aurium; tongue coated; some nausea; discharges slightly less frequent, but still contain blood and mucus, mixed with bile; tenesmus same as yesterday; ordered treatment continued, with the addition of hydrarg. chloridi mite gr. i., to each powder.

July 16.—Patient passed a more comfortable night than since the attack; seems less irritable; pain in bowels subsiding; tongue slightly improving in appearance; discharges frequent, but not so offensive; no appetite; calomel omitted; salicine continued.

July 17.—Passed comfortable night and much improved; appetite slowly returning; able to sit up in bed; tongue clean; discharges less frequent; pain and tenesmus almost nil; pulse and temperature normal; salicine continued "ter in die."

July 18.—Patient slept well all night; had no discharge from bowels since previous evening; pain, etc., entirely relieved; appetite good; ate a hearty breakfast and feels well; discharged.

No comment is needed, and a repetition of cases unnecessary. A trial of the drug will generally lead to its extended use; for, in my own hands, it has certainly proven almost a specific.

---

Extract of Conium, in doses of 2 grs., repeated several times a day, is said by some to be a valuable remedy in preventing inflammation of the breast of recently confined women, as a consequence of over-distinction of the milk ducts.

